# Supplementary material for: Implicit Memory and Anesthesia: A Systematic Review and Meta-Analysis
Source: Life (Basel). 2021 Aug 19;11(8):850. doi: 10.3390/life11080850 (PMC8400596; doi:10.3390/life11080850)
Supplement: Supplementary file 1 [file life-11-00850-s001.zip › life-1330508-supplementary.pdf]

Table S1. Caracteristic of considered trials.

Study	Type of Surgery	Type of Anaesthesia	Anaesthetic Regimen	Pre-medication	Light Anaesthesia	Light Analgesia	ABM	NMBA	Anaesthesia >60 min	Patients (N)	Test adopted	Surgical Stimulation during Word presentation	Words Presented Throughout Anaesthesia	Implicit Memory
Millar and Watkinson, 1983	Gynaecological - General	General	Induction: IV Maintenance: IA	Yes/No	Yes	Yes	No	Yes	Nd	27	WRT	Yes	No	Yes
Jelicic et al., 1990	Nd	General	Induction: IV Maintenance: IA	Nd	Yes	No	No	Yes	Nd	25	FAT	Yes	No	Yes
Kihlstrom et al., 1990	Gynaecological - Orthopaedic - Abdominal - Vascular	General	Induction: IV Maintenance: IA	No	No	Yes	No	Yes	Yes	25	WRT - FAT	Yes	Yes	Yes
Block et al., 1991	Gynaecological - General	General	Induction: IV Maintenance: IA	No	Yes/No	Yes/No	No	Yes	Yes	56	WSC - WRT	Yes	Yes/No	Yes
Bethune et al., 1992	Cardiac -	General	Induction: IV Maintenance: IV	No	Yes	No	No	Yes	Yes	19	FAT	Yes	Yes	Yes/No
Brown et al., 1992	Ophthalmic	General	Induction: IV Maintenance: IA	No	No	No	No	Yes	Nd	10	CG - SGT	No	No	Yes
Dwyer et al., 1992	General	General	Induction: IV Maintenance: IA	No	No	Yes	No	Yes	Yes	90	GK	Yes/No	No	No
Jelicic et al., 1992a	Ophthalmic	General	Induction: IV Maintenance: IA	No	Yes	No	No	Yes	Nd	43	GK	Yes	No	Yes
Jelicic et al., 1992b	Nd	General	Induction: IV Maintenance : IA	Nd	Yes	No	No	Yes	Nd	25	CG	Yes	No	Yes
Cork et al., 1992	Nd	General	Induction: IV Maintenance: IA	No	Yes	No	No	Yes	Yes	25	FAT - WR	Yes	Yes	No
Bonebakker at al., 1993	Elective	General	Induction: IV Maintenance: IA	No	No	No	No	Yes	Nd	81	CG	Yes	No	No
Jelicic et al., 1993	Body surface	General	Induction: IV Maintenance: IA	No	No	Yes	No	No	Nd	41	GK	Yes	No	No
Munglani et al., 1993	Volunteers	Sedation	Induction: IA Maintenance: IA	No	Yes	Yes	No	No	Nd	9	FCR	No	No	No
Westmoreland et al., 1993	Nd	General	Induction: IV Maintenance: IA	Yes/No	No	No	No	Yes	Nd	96	FAT - CG	Yes	Yes	No
Parker et al., 1994	Minor or intermediate	General	Induction: IV Maintenance: IA	Yes	Yes	Yes	No	Yes	Yes	24	FAT	Yes	No	No
Schwender et al., 1994	Cardiac	General	Induction: IV Maintenance: IA/IV	Yes	Yes/No	Yes	No	Yes	Yes	30	FAT	Yes	No	Yes
De Roode et al., 1995	Ophthalmic	General	Induction: IV Maintenance: IA/IV	Yes	Yes	Yes	No	Yes	Nd	83	GK - FCR	Yes	No	No
Gonsowski et al., 1995	Volunteers	General	Induction: IV Maintenance: IA	No	Yes	Yes	No	Yes	Yes	12	FAT - GK	No	No	No
Van Hooff et al., 1995	Cardiac	General	Induction: IV Maintenance: IV	Yes/No	No	No	No	Yes	Yes	9	CG - WRT	Yes	No	Yes
Bonebakker at al., 1996	Gynecological - General - Plastic -	General	Induction: IV Maintenance: IA	No	No	No	No	Yes	Nd	489	WSC - FCR	Yes	No	Yes/No

	Orthopaedic - Traumatology													
Donker et al., 1996	Orthopedic	General	Induction: IV Maintenance: IV	No	Yes	No	No	Yes	No	58	GK	Yes	No	No
Russell and Wang, 1997	Major - Gynaecological	General	Induction: IV Maintenance: IA	Yes	No	Yes	No	Yes	No	38	CG - FAT	Yes	Yes	No
Struys et al., 1998	Gynaecological	Sedation	Induction: IV Maintenance: IV	No	No	Yes	Yes	No	No	58	FAT	Yes	No	No
Münte et al., 1999	Vertebral	General	Induction: IV Maintenance: IA+IV	Yes	No	No	No	Yes	Yes	60	WSC - **FFJ**	Yes	No	Yes/No
Lubke et al., 1999	Orthopaedic - Traumatology	General	Induction: IV Maintenance: IA	No	No	No	Yes	Yes	Yes	96	WSC with PDP	Yes	No	Yes
De Roode et al., 2000	Volunteers	Sedation	Induction: IV Maintenance: IV	No	Yes/No	Yes	No	No	Yes	20	WSC	No	No	No
Ghoneim et al., 2000	Gynaecologic-Orthopedic - General - Plastic	General	Induction: IV Maintenance: IA	No	No	Yes/No	No	Yes	Yes	179	FAT	Yes	Yes	Yes/No
Lubke et al., 2000	Caesarean section	General	Induction: IV Maintenance : IA	No	No	Yes	Yes	Yes	No	24	WSC with PDP	Yes	No	Yes
Münte et al., 2000	Vertebral	General	Induction: IV Maintenance: IA	Yes	No	No	No	Yes	Yes	30	FFJ	Yes	No	No
Stapleton et al., 2000	Minor - gynaecological	Sedation	Induction: IV Maintenance: IV	No	Yes	Yes	No	No	No	32	WSC with PDP	Yes	No	No
Renna et al., 2000	Minor - gynaecological	Sedation	Induction: IA Maintenance: IA	No	Yes	Yes	Yes	No	Nd	47	WRT	No	No	Yes/No
Andrade et al., 2001	Laparoscopic ?	General	Induction: IA Maintenance: IA+IV	No	No	Yes	Yes	Yes	Nd	24	CG	No	No	No
Gidron et al., 2002	Laparoscopic cholecystectomy	General	Induction: IV Maintenance: IA	No	No	No	No	Yes	Nd	30	FAT	Yes	Nd	Yes
Münte et al., 2001	ICD implantation	General	Induction: IV Maintenance: IV	Yes	Yes	No	No	Yes	Yes	30	**FFJ**	Yes	No	No
Kerssens et al., 2001	Orthopaedic - General - Urologic	General	Induction: IV Maintenance: IV	No	No	Yes	Yes	Yes	No	41	WRT - CG	Yes	No	No
Loveman et al., 2001	Cardiac	General	Induction: IV Maintenance: IV	Yes	Yes	Yes	No	Yes	No	14	WSC - FCR	No	Nd	No
Russell and Wang, 2001	Major - gynaecological	General	Induction: IV Maintenance: IV	Yes	No	No	No	Yes	Yes	40	CG - WRT	Yes	Yes	No
Münte et al., 2002	Vertebral	General	Induction: IV Maintenance: IA+IV	Yes	No	No	No	Yes	Yes	60	WRT - FFJ	Yes	No	No
Kerssens et al., 2002	General – Orthopaedic - Plastic	General	Induction: IV Maintenance: IV	No	Yes	Yes	Yes	Yes	Yes	56	WSC with PDP	No	Nd	Yes
Lequeux et al., 2003	Minor general surgery	General	Induction: IV Maintenance: IV	No	No	Yes	No	Yes	Yes	20	WSC - FCR	No		No

Münte et al., 2003	Laparoscopic inguinal herniotomy	General	Induction: IV Maintenance: IV	No	No	No	Yes	Yes	Yes	128	FFJ	Yes/No	Yes/No	Yes/No
Deeprose et al., 2005	Orthopaedic	General	Induction: IV Maintenance: IA	No	No	Yes	Yes	No	No	126	WSC	Yes/No	No	Yes
Lequeux et al., 2005	Minor	Sedation	Induction: IV Maintenance: IV	No	Yes	No	Yes	No	Nd	10	WSC - FCR	No	Nd	No
Kerssens et al., 2005	Orthopaedic - General	General	Induction: IV Maintenance: IA+IV	No	No	No	Yes	Yes	Yes	90	WSC with PDP	Yes	No	No
Stonell et al., 2006	Nd	General	Induction: IV Maintenance: IA	No	No	Yes	Yes	Yes	Yes	113	WSC with PDP	Yes	Nd	Yes
Wang et al., 2006	Lower limb	Sedation	Induction: IV Maintenance: IV	Nd	Yes	No	Yes	No	Nd	12	WSC with PDP	Yes	Nd	No
Dobrunz et al., 2007	Gynaecological	General	Induction: IV Maintenance: IA	Yes/No	No	No	Yes	Yes	Yes	120	WRT - WSC	Yes/No	No	Yes/No
Bejjani et al., 2009	Cardiac	General	Induction: IV Maintenance: IV	Yes	No	No	Yes	Yes	Yes	38	WSC	Yes	No	No
Hadzidiakos et al., 2009	Urological – Gynaecological – General - Orthopedic	General	Induction: IV Maintenance: IV	Yes	No	No	Yes	Yes	Yes	119	WSC with PDP	Yes	Yes	No
Kerssens et al., 2009	Major - Orthopaedic	General	Induction: IV Maintenance: IA	No	No	No	Yes	Yes	Yes	109	WRT	Yes	No	Yes/No
Tian et al., 2010	Volunteers	Sedation	Induction: IV Maintenance: IV	No	Yes	Yes	No	No	No	6	WSC with PDP	No	Nd	No
Ozcan et al., 2011	Orthopaedic	General	Induction: IV Maintenance: IA	Yes	No	No	Yes	Yes	Yes	37	WSC	Yes	No	No
Liu et al., 2012	Volunteers	Sedation	Induction: IV Maintenance: IV	No	No	Yes	No	No	Nd	8	FCR	No	Nd	No
Flouda et al., 2013	Elective	General	Induction: IV Maintenance: IA/IV	No	Yes	No	Yes	Yes	Yes	93	WSC with PDP	Yes	Yes	Yes
Quan et al., 2013	Volunteers	Sedation	Induction: IV Maintenance: IV	No	Yes	Yes	Yes	No	Yes	12	WSC with PDP	No	Nd	No
Lequeux et al., 2014	Nd	General	Induction: IV Maintenance: IV	Yes	No	No	Yes	Yes/No	Yes	78	WSC - FCR	Yes	Yes	No
Levantesi et al., 2016	Major abdominal	General	Induction: IV Maintenance: IA	No	No	No	Yes	Yes	Nd	64	FAT	Yes	No	Yes/No
Elbadawy et al., 2015	Orthopaedic	General	Induction: IV Maintenance: IA/IV	No	No	Yes	Yes	Yes	Yes	400	WRT	Yes	No	No
Xin-de Chen et al., 2018	Orthopaedic	General	Induction: IV Maintenance: IA/IV	No	No	Yes	Nd	Yes	Yes	80	WRT	Yes	No	Yes
Kallionpää et al., 2018	Volunteers	Sedation	Induction: IV Maintenance: IV	No	Yes	Yes	No	No	No	47	FFJ	No	Nd	No
Aceto et al., 2003	Laparoscopic cholecystectomy	General	Induction: IV Maintenance:IA	No	No	No	No	Yes	Yes	40	FAT	Yes	No	No

Psychological tests for implicit memory detecting: category generation (CG), sentence generation task (SGT), free association test (FAT), word stem completion test (WSC), word recognition test (WRT), general knowledge (GK), forced-choice recognition (FCR), preference, familiarity fame judgements (FFJ), process dissociation procedure (PDP). NMBA: neuromusular blocking agent; ICD: implantable cardioverter Defibrillator; IV: Intravenous Anaesthesia; IA: Inhalational Anaesthesia.

Table S2. Distribution of cohorts among the considered variables during only general anaesthesia regimens.

ASA: American Society of Anesthesiologist physical status classification; NMBA:

**Variable**	**Implicit Memory**					
	**Yes**	**No**	**OR**	**L95%CI**	**U95%CI**	**P-value**
**Age**						
≤ 50 years	31	44				
> 50 years	11	10	1.61	0.61	4.27	0.47
**ASA**						
I-II	23	44				
III-IV	10	7	2.73	0.92	8.13	0.12
**Duration**						
≤ 60 minutes	4	9				
> 60 minutes	25	37	1.52	0.42	5.48	0.74
**Premedication**						
No benzodiazepines	33	38				
Benzodiazepines	7	23	0.35	0.13	0.92	**0.05**
**Induction**						
No benzodiazepines	39	57				
Benzodizepines	3	4	1.10	0.15	6.87	1.0
**Maintenance**						
Intravenous	28	38				
Inhalational	14	22	1.13	0.49	2.58	0.94
No N_2O during maintenance	20	31				
N_2O during maintenance	22	30	1.14	0.52	2.50	0.91
No benzodiazepines	41	61				
Benzodiazepines	1	0	inf	0.03	inf	0.41
No opioids	5	10				
Opioids	37	51	1.45	0.46	4.60	0.73
No NMBA	3	3				
NMBA	39	58	0.68	0.27	1.79	0.69
No light anaesthetic regimen	29	49				
Light anaesthetic regimen	13	12	1.83	0.74	4.54	1.0
No deep anaesthetic regimen	13	13				
Deep anaesthetic regimen	29	48	0.60	0.25	1.48	0.38
No light analgesic regimen	25	39				
Light analgesic regimen	17	22	1.21	0.54	2.70	0.81
No deep analgesic regimen	17	24				
Deep analgesic regimen	25	37	0.95	0.43	2.13	1.0
**Monitoring**						
No AMB-guided anaesthesia	35	44				
ABM-guided anaesthesia	7	16	0.55	0.20	1.49	0.34
**Listening to the auditory task**						
No during surgical stimulation	4	8				
During surgical stimulation	38	53	1.43	0.35	6.97	0.76
No during all the maintenance period	30	44				
During all the maintenance period	7	16	0.64	0.24	1.75	0.53
**Timing of memory testing**						
≤ 24 hours	30	41				
> 24 hours	12	20	0.82	0.35	1.93	0.81

neuromuscular blocking agent; ABM: anaesthesia brain monitor; OR: odds ratio; L95%CI and U95%CI: lower limit and upper limit of the 95% confidence interval (CI).

Table S3. Distribution of cohorts among the considered variables during only inhalational general anaesthesia regimens.

Variable	Implicit memory					
	**Yes**	**No**	**OR**	**L95%CI**	**U95%CI**	**P-value**
**Age**						
≤ 50 years	22	28				
> 50 years	5	5	1.27	0.33	4.96	1.0
**ASA**						
I-II	14	30				
III-IV	7	5	3.00	0.81	11.13	0.18
**Duration**						
≤ 60 minutes	4	9				
> 60 minutes	25	37	1.21	0.06	76.85	1.0
**Premedication**						
No benzodiazepines	23	26				
Benzodiazepines	3	13	0.26	0.07	1.03	**0.09**
**Induction**						
No benzodiazepines	39	57				
Benzodizepines	3	1	0	0	54.28	1.0
**Maintenance**						
No N_2O during maintenance	10	14				
N_2O during maintenance	18	25	1.01	0.37	2.78	1.0
No opioids	4	7				
Opioids	24	32	1.31	0.29	6.81	0.75
No NMBA	3	2				
NMBA	39	37	inf	0.14	inf	0.51
No light anaesthetic regimen	29	32				
Light anaesthetic regimen	13	7	2.17	0.69	6.77	0.29
No deep anaesthetic regimen	9	8				
Deep anaesthetic regimen	19	31	0.55	0.18	1.66	0.43
No light analgesic regimen	25	39				
Light analgesic regimen	17	22	0.76	0.27	2.11	0.78
No deep analgesic regimen	9	17				
Deep analgesic regimen	19	22	0.63	0.59	4.50	0.49
**Monitoring**						
No ABM monitoring	19	30				
ABM monitoring	7	9	1.23	0.39	3.85	0.95
No AMB-guided anaesthesia	24	31				
ABM-guided anaesthesia	4	8	0.65	0.17	2.40	0.74
**Listening to the auditory task**						
No during surgical stimulation	1	4				
During surgical stimulation	27	35	3.04	0.28	157.37	0.39
No during all the maintenance period	21	29				
During all the maintenance period	5	10	0.69	0.21	2.32	0.76
**Timing of memory testing**						
≤ 24 hours	19	22				
> 24 hours	9	17	0.61	0.22	1.69	0.49

ASA: American Society of Anesthesiologist physical status classification; NMBA: neuromuscular blocking agent; ABM: anaesthesia brain monitor; OR: odds ratio; L95%CI and U95%CI: lower limit and upper limit of the 95% confidence interval (CI).

Table S4. Distribution of cohorts among the considered variables during only intravenous general anaesthesia regimens.

Variable	Implicit memory					
	Yes	No	OR	L95%CI	U95%CI	P-value
**Age**						
≤50 years	8	16				
>50 years	6	5	2.40	0.56	10.32	0.41
**ASA**						
I-II	9	14				
III-IV	3	2	2.33	0.32	16.82	0.72
**Duration**						
≤ 60 minutes	3	7				
> 60 minutes	11	14	1.80	0.31	13.35	0.70
**Praemedication**						
No benzodiazepines	10	12				
Benzodiazepines	4	10	0.48	0.12	2.01	0.51
**Induction**						
No benzodiazepines	11	19				
Benzodizepines	3	3	1.70	0.19	15.04	0.66
**Maintenance**						
No N_2O during maintenance	10	17				
N_2O during maintenance	4	5	1.35	0.21	8.04	0.71
No benzodiazepines	13	1				
Benzodiazepines	1	0	inf	0.04	inf	0.39
No opioids	1	3				
Opioids	13	19	2.02	0.14	115.84	1.0
No NMBA	3	1				
NMBA	11	21	0.19	0.00	2.59	0.28
No light anaesthetic regimen	29	32				
Light anaesthetic regimen	13	7	1.35	0.21	8.04	0.71
No deep anaesthetic regimen	4	5				
Deep anaesthetic regimen	10	17	0.74	0.12	4.67	0.71
No light analgesic regimen	6	15				
Light analgesic regimen	8	7	2.86	0.71	11.44	0.25
No deep analgesic regimen	8	7				
Deep analgesic regimen	6	15	0.35	0.09	1.40	0.25
**Monitoring**						
No ABM monitoring	5	10				
ABM monitoring	7	12	1.16	0.28	4.84	1.0
No AMB-guided anaesthesia	11	13				
ABM-guided anaesthesia	3	8	0.45	0.06	2.52	0.46
**Listening to the auditory task**						
No during surgical stimulation	3	4				
During surgical stimulation	11	18	0.82	0.11	6.68	1.0
No during all the maintenance period	9	15				
During all the maintenance period	2	6	0.57	0.05	4.14	0.68
**Timing of memory testing**						
≤ 24 hours	11	19				
> 24 hours	3	3	1.70	0.19	15.04	0.66

ASA: American Society of Anesthesiologist physical status classification; NMBA: neuromuscular blocking agent; ABM: anaesthesia brain monitor; OR: odds ratio; L95%CI and U95%CI: lower limit and upper limit of the 95% confidence interval (CI).
